# PET Waste Recycling into BTX Fraction Using In Situ Obtained Nickel Phosphide

**DOI:** 10.3390/polym15102248

**Published:** 2023-05-10

**Authors:** Maria Golubeva, Mariyam Mukhtarova, Alexey Sadovnikov, Anton Maximov

**Affiliations:** A.V.Topchiev Institute of Petrochemical Synthesis, Russian Academy of Sciences (TIPS RAS), Moscow 119991, Russia

**Keywords:** PET, polyethylene terephthalate, plastic waste, waste recycling, nickel phosphide, Ni_2_P, BTX, dispersed catalysts

## Abstract

The annual production of plastic waste is a serious ecological problem as it causes substantial pollution of the environment. Polyethylene terephthalate, a material usually found in disposable plastic bottles, is one of the most popular material used for packaging in the world. In this paper, it is proposed to recycle polyethylene terephthalate waste bottles into benzene-toluene-xylene fraction using a heterogeneous nickel phosphide catalyst formed in situ during the polyethylene terephthalate recycling process. The catalyst obtained was characterized using powder X-ray diffraction, high-resolution transmission electron microscopy, and X-ray photoelectron spectroscopy techniques. The catalyst was shown to contain a Ni_2_P phase. Its activity was studied in a temperature range of 250–400 °C and a H_2_ pressure range of 5–9 MPa. The highest selectivity for benzene-toluene-xylene fraction was 93% at quantitative conversion.

## 1. Introduction

More than 380 million tons of plastic are produced annually in the world [1]. However, in 2015 it was reported that more than 50% of plastics are never recycled in any way. Some plastic waste ends up in landfills, and some goes into the environment, thereby polluting it and having a detrimental effect on the inhabitants. Under normal environmental conditions, depending on the type, plastic decomposes within 10–1000 years, and on average, its decomposition time is measured in hundreds of years.

One of the most common types of plastic is polyethylene terephthalate (PET), which is widely used across the world to make bottles for water and soft drinks, textile fibers, sheets, and films [2,3]. Only a part of PET bottles is recycled into fiber, and is rarely reused for bottle production [4]. This type of recycling is called mechanical, which is the most common type of plastic recycling. However, mechanical recycling requires careful sorting of waste for the selection of the same composition plastics [5]. Moreover, a major problem in PET mechanical recycling is the fact that mechanical properties of recycled PET (rPET) decline significantly [6].

Chemical recycling is an alternative method for PET processing. Since PET contains an aromatic structure, not only the PET monomer, and terephthalic acid (TPA), but also arenes, such as benzene, toluene and xylenes, can be obtained. *p*-Xylene is known to be a raw material for TPA production [7]. Thus, PET can be recycled back into its raw material. Heterogeneous catalysts are effective for this goal [8]. For instance, Jing et al. made an investigation on various noble metal-based catalyst activity in PET recycling into aromatics (toluene + *p*-xylene) [9]. Ru-based catalysts were shown to be more active and selective than Pt- and Pd-based catalysts. Ru-based catalyst activity decreased in a row: Ru/Nb_2_O_5_ > Ru/ZrO_2_ > Ru/TiO_2_ > Ru/HZSM-5 (200 °C, 0.3 MPa H_2_, 12 h). The yield of aromatics over Ru/Nb_2_O_5_ was 80.1%. In another work of this research group, a comparison of Pt/NiAl_2_O_4_, Pd/NiAl_2_O_4_, Ru/NiAl_2_O_4_, and Ru/Nb_2_O_5_ activity in H_2_-free conversion of PET to benzene-toluene-xylenes (BTX) fraction was conducted [10]. H_2_ was obtained using aqueous-phase reforming of ethylene glycol after PET depolymerization. The BTX yield decreased after using catalysts in the following order: Ru/Nb_2_O_5_ > Ru/NiAl_2_O_4_ > Pd/NiAl_2_O_4_ > Pd/NiAl_2_O_4_ (220 °C, 2 MPa N_2_, 12 h). Ru/Nb_2_O_5_ provided the BTX yield of 91.3%.

A three-step process of PET recycling was considered by Tang et al. [11]. In the first step, thermal alcoholysis of PET was carried out. Methanol demonstrated the highest effectiveness in alcoholysis compared to ethanol and butanol. Dimethyl terephthalate (DMT) yield was 97.3% at 200 °C, after 3.5 h of reaction. In the second step, DMT was hydrogenated to dimethyl cyclohexane-1,4-dicarboxylate (DMCD) over Pt/C, Ru/C or Pd/C. In the third step, DMCD was hydrodeoxygenated over Ru-Cu/SiO_2_ catalyst, with C_7_–C_8_ cycloalkanes and aromatics being observed. The yield of aromatics was 38% (400 °C, 4 MPa H_2_; DMCD flow rate: 0.04 mL·min^−1^, and H_2_ flow rate: 120 mL·min^−1^).

Hongkailers et al. carried out an investigation on Co/TiO_2_ activity in PET recycling into arenes under H_2_ pressure [12]. At first, TPA was used as a substrate. The yield of arenes, such as *p*-xylene and toluene, was 75.2 mol. %. (340 °C, 3 MPa H_2_, 4 h). Then, the investigation of PET recycling was carried out. Co/TiO_2_ catalyst was shown to promote both the depolymerization of PET and the hydrodeoxygenation (HDO) of monomers obtained. Toluene and *p*-xylene were both observed with 78.9 mol. % yield. Thereafter, the possibility of Co/TiO_2_ reusing was investigated. After the recycle, the total product yield decreased from 90 mol. % to 32 mol. %, while arene yield decreased from 69 mol. % to 4 mol. %. Catalyst deactivation was related to CoTiO_3_ phase formation instead of individual phases of Co and TiO_2_.

The present study sets out the recycling of waste PET bottle into BTX fraction using in situ obtained nickel phosphide. Recently, nickel phosphide showed high activity in the HDO of various oxygen-containing substrates [13]. Moreover, it is an approach to the in situ synthesis of nickel phosphide-based catalysts in the reaction medium that we suggested [14,15,16]. Nickel phosphide-based catalysts were obtained from water- and oil-soluble precursors in polar or non-polar medium during the HDO of biomass-derived compounds, such as furfural [14], guaiacol [15], and levulinic acid [16]. Partially and fully deoxygenated products were obtained. This approach was applied in the present work. In contrast to the previous works, which used two different precursors containing nickel and phosphorus separately, a catalyst precursor used in the present work contained both nickel and phosphorus in its composition. This allows to fix the Ni/P ratio throughout all catalytic experiments. Nickel phosphide-based catalyst was obtained from the Ni-P-containing precursor during the depolymerization and the HDO of PET. The highest selectivity of BTX fraction was 93 wt. % at quantitative PET conversion over in situ generated nickel phosphide-based catalyst (400 °C, 9 MPa H_2_, 6 h).

## 2. Materials and Methods

### 2.1. Synthesis of Nickel Phosphide Precursor

A total of 5 g of nickel acetate tetrahydrate (98%, Sigma-Aldrich, St. Louis, MO, USA), 3.9 g of hypophosphorous acid (50 wt. % in water, Sigma-Aldrich, St. Louis, MO, USA), and 100 mL of deionized water were mixed in a 150 mL glass cup. The mixture was stirred while heating to dissolve the reagents and then slowly evaporate water. A greenish-yellow precipitate was formed. The water residues were removed in an oven at 120 °C for 3 h. After that, the solid was ground in a porcelain mortar to obtain a powder.

### 2.2. Catalytic Tests

The polyethylene terephthalate bottle used for storing sparkling mineral water was milled. The fraction containing particles of ~0.04 cm^2^ in size was selected for the experiment. A total of 150 mg of polyethylene terephthalate, 90.7 mg of the precursor, 1.5 g of dodecane (Sigma-Aldrich, St. Louis, MO, USA, >99%), and a magnetic stirrer were put in a batch autoclave reactor of 45 cm^3^ in volume. The reactor was sealed, filled with hydrogen (≥98%, Air Liquide, Paris, France) up to 5–9 MPa and heated to 250–400 °C while stirring. The catalytic reaction was carried out for 6 h. Then, the reactor was cooled to room temperature and depressurized. The reaction products were separated from the catalyst via centrifugation (5000 rpm). The catalyst was washed several times using dimethyl sulfoxide (DMSO; Component-reaktiv, Moscow, Russia, >99.8%) to remove terephthalic acid, and also washing using petroleum ether 40/70 (Component-reaktiv, Moscow, Russia, tech.). Then, the catalyst was dried at room temperature in atmospheric argon (≥98%, Air Liquide, Paris, France) until the ether completed evaporation, then ground in a porcelain mortar and passed through the sieve (mesh size of 0.2 mm) to remove PET leftovers.

### 2.3. Characterization

The phase composition of the catalysts obtained was determined using powder X-ray diffraction (XRD). The diffractograms were obtained in a range of 10−60° 2θ using a Rigaku Rotaflex D/MAX-RC and a Bruker D8 Advance diffractometers containing CuK_α_ radiation. Qualitative phase analysis of the samples was carried out using the PDF-2 ICDD database of powder diffraction patterns. The average sizes of crystallites were calculated using the Scherrer equation.

Transmission electron microscopy (TEM), high-resolution transmission electron microscopy (HRTEM) images and elemental maps of the catalyst samples were obtained using a FEI Tecnai Osiris transmission electron microscope equipped with a field emission electron gun operated at 200 kV and an attachment for energy dispersive X-ray (EDX) spectroscopy analysis. Fast Fourier transformation (FFT) of the HRTEM images obtained was carried out using Gatan DigitalMicrograph software.

X-ray photoelectron spectra (XPS) of the catalyst were obtained using a PREVAC EA15 electronic spectrometer and AlK_α_ radiation source (hν = 1486.74 eV, 150 W). Spectra deconvolution was carried out using a PeakFit software.

Gaseous carbon-containing products were identified using a Chromos GH-1000 chromatograph equipped with a thermal conductivity detector, and two packed columns: a NaX/3X molecular sieve column (2 m × 3 mm), and a HayeSep R column (3 m × 3 mm), with helium used as a carrier gas.

Terephthalic acid obtained after the catalytic reaction was identified using NMR. ^1^H and ^13^C NMR-spectra of TPA were recorded on a Bruker AVANCE III HD (400 MHz) spectrometer, with a solvent signal used as internal reference. DMSO-d_6_ (Solvex-D, 99.8%) was applied to dissolve TPA.

The qualitative analysis of liquid reaction products was carried out via gas chromatography–mass spectrometry (GC–MS) using a Thermo Scientific ISQ 7000 GC–MS equipped with a Restek 5XI-17SIL MS CAP capillary column (30 m × 0.25 mm × 0.25 μm), with helium as a carrier gas. The quantitative analysis of liquid reaction products was carried out via gas–liquid chromatography (GLC) using a Crystallux 4000 M gas chromatograph equipped with a flame ionization detector, an Optima-1 capillary column (25 m × 0.32 mm × 0.35 μm), and with helium used as a carrier gas. *PET conversion* (wt. %) and *product selectivity* (%) were calculated using the following equations:

(1) $$PET\;conversion\;\left(wt.\;\%\right)=\frac{initial\;mass\;of\;PET-mass\;of\;unreacted\;PET}{initial\;mass\;of\;PET}\times 100\%,$$

(2) $$Product\;selectivity\;\left(\%\right)=\frac{mass\;of\;product\;formed}{\sum mass\;of\;all\;products}\times 100\%.$$

## 3. Results and Discussion

### 3.1. Nickel Phosphide In Situ Formation

Recently, we studied the in situ formation of nickel phosphide catalysts during the hydrodeoxygenation of oxygen-containing bio-based substrates using hypophosphorous acid and nickel (II) acetate as catalyst precursors [14,15,16]. In this work, a solid catalyst precursor, nickel (II) hypophosphite, was easily obtained from the compounds mentioned above. It was conducted in order to avoid even a minimal variation in the acid/salt ratio, and the presence of free acid and water in the reaction medium at the initial moment of reaction.

As a result of nickel (II) acetate and hypophosphorous acid mixture heating in an aqueous solution, nickel (II) acetate was hydrolyzed to form nickel (II) hydroxide, which was dissolved in hypophosphorous acid to form nickel hypophosphite. Then, the formation of nickel phosphide from nickel hypophosphite during the decomposition of PET under H_2_ pressure has been investigated.

**Figure 1:**
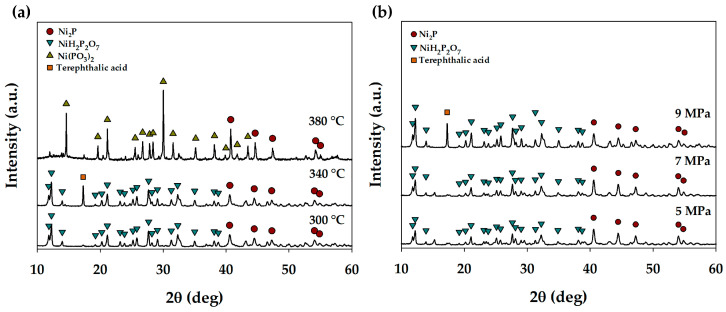
XRD patterns of the catalyst samples obtained at (**a**) 300, 340, 380 °C, 9 MPa H_2_, 6 h; (**b**) 340 °C, 5, 7, 9 MPa H_2_, 6 h.

The effect of the reaction temperature (300, 340, and 380 °C) and pressure (5, 7, and 9 MPa H_2_) on the formation of nickel phosphide was studied using XRD (Figure 1). The nickel phosphide phase of Ni_2_P (PDF №74-1385) was shown to be formed in all cases. Ni_2_P reflections were identified in the samples at 2θ = 40.6°, 44.5°, 47.2°, 54.0°, 54.8° and corresponded to the (111), (201), (210), (300), (211) planes, respectively. In addition to the Ni_2_P phase, phosphate phases were formed. NiH_2_P_2_O_7_ (PDF №34-617) was identified in the samples obtained at 300 °C, 9 MPa H_2_; and at 340 °C, 5, 7, 9 MPa H_2_. Phosphates can be formed as a result of hypophosphite disproportionation. Guan et al. [17] supposed that the following reaction occurs:

Ni(H_2_PO_2_)_2_ = PH_3_↑ + NiHPO_4_. (3)

Then, NiHPO_4_ can be transformed into NiH_2_P_2_O_7_ when heated [18,19]. In the sample of the catalyst obtained at 380 °C under 9 MPa H_2_, the phase of Ni(PO_3_)_2_ (PDF №28-708) was found as a result of NiH_2_P_2_O_7_ decomposition at higher temperature [19]. Moreover, in the sample obtained at 340 °C under 9 MPa H_2_, terephthalic acid (PDF №31-1916) was identified. This indicates the formation of TPA during the decomposition of PET over nickel phosphide, and also that the sample was not completely washed. Thus, the reaction conditions do not significantly affect the phase composition of the catalysts; however, with an increase in the reaction temperature, NiH_2_P_2_O_7_ decomposes with the formation of Ni(PO_3_)_2_.

The average crystallite sizes of the phases obtained are presented in Table S1. With an increase in reaction temperature, crystallite size enlargement of Ni_2_P is observed, which may be due to particle sintering at a higher temperature. For phosphate phases, a similar dependence is observed. The dependence of the crystallite sizes on the initial H_2_ pressure is not found; the difference in sizes is within the error for phosphide as well as for phosphate.

**Figure 2:**
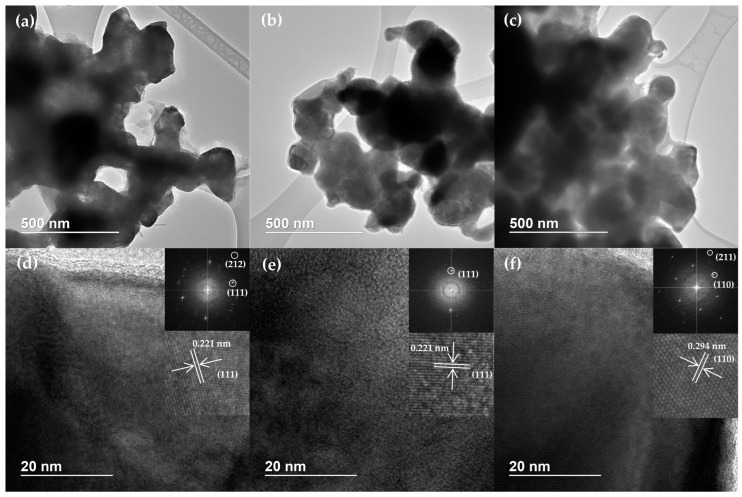
TEM, HRTEM images and corresponding reduced FFT of Ni_2_P-containing catalysts obtained in situ under 9 MPa H_2_ at (**a**,**d**) 300 °C; (**b**,**e**) 340 °C; (**c**,**f**) 380 °C.

Since the effect of initial pressure on the catalyst phase composition and crystallite sizes was negligible, only the samples obtained at different temperatures were further studied. The morphology of Ni_2_P-containing catalysts obtained in situ at 300–380 °C, under 9 MPa H_2_, during 6 h was studied using TEM (Figure 2a–c). HRTEM images of the catalysts (Figure 2d–f) show a crystal lattice with interplanar spacings corresponding to Ni_2_P. Due to a reduced FFT of HRTEM images, diffraction patterns of the catalysts were obtained. There are reflections of (111) and (212) planes in Ni_2_P with interplanar spacings of 0.221 nm and 0.128 nm, respectively, in the diffraction pattern of the sample obtained at 300 °C (Figure 2d). The diffraction pattern of the sample obtained at 340 °C shows reflections corresponding to (111) plane of Ni_2_P (Figure 2e). The reflections in the diffraction pattern of the sample obtained at 380 °C are attributed to (110) and (211) planes in Ni_2_P with interplanar spacings of 0.294 nm and 0.167 nm, respectively (Figure 2f). Thus, the presence of the Ni_2_P phase in the catalyst samples, identified using the XRD, is confirmed via the HRTEM technique.

Ni and P elemental maps of the catalysts (Figure S1) indicate the uniform distribution of these elements in the samples. Oxygen as a part of phosphates was also identified.

**Figure 3:**
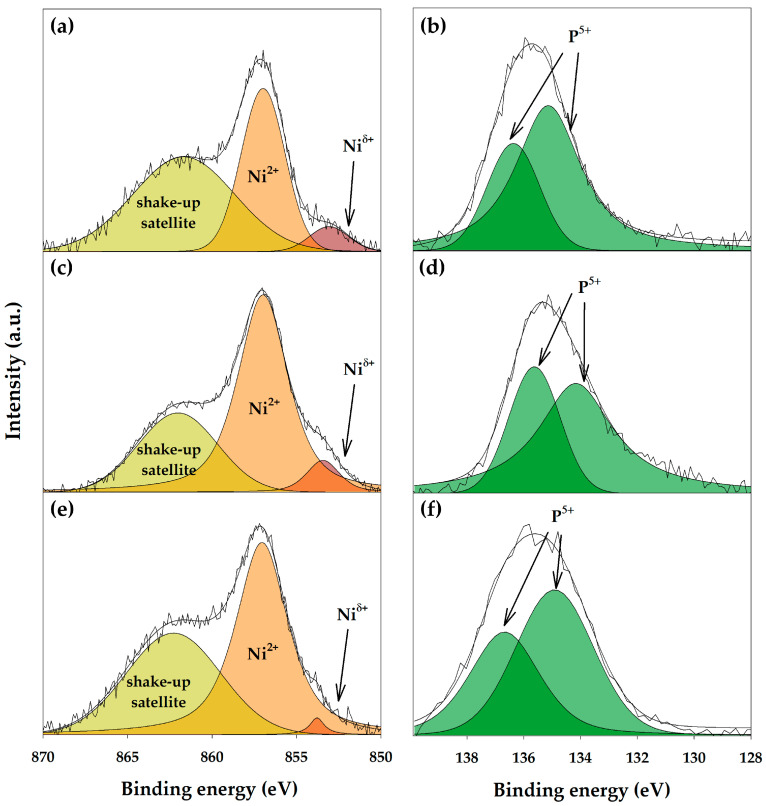
XPS in Ni2*p*_3/2_ and P2*p* regions for the Ni_2_P-containing catalysts obtained in situ under 9 MPa H_2_ at (**a**,**b**) 380 °C; (**c**,**d**) 340 °C; (**e**,**f**) 300 °C.

The electronic states on the Ni_2_P surface were studied using XPS. The Ni2*p*_3/2_ spectra are shown in Figure 3a,c,e. Each spectrum was deconvoluted into three peaks. The peaks with a maximum at 853.1–853.8 eV are ascribed to Ni^δ+^ species and associated with Ni_2_P state [20]. The peaks at 856.7–857.0 eV are corresponded to Ni^2+^ species in nickel phosphate. The binding energies of 861.7–862.3 eV are related to the shake-up satellite peaks accompanying the Ni^2+^ main peaks. The content of Ni^2+^ species on the catalyst surface exceeds the content of Ni^δ+^, which can be associated with the surface oxidation of the catalysts, as well as the incomplete reduction of phosphate formed by the decomposition of the precursor. The P2*p* spectra are presented in Figure 3b,d,f. The binding energy values of 134.2–135.2 eV and 135.7–136.4 eV are corresponded to P^5+^ in 2*p*_3/2_ and 2*p*_1/2_ regions, respectively. P^δ−^ species were not identified on the catalyst surface.

The presence of Ni^δ+^ species leads to the formation of metal active sites, while the presence of Ni^2+^ and P–OH leads to the formation of Lewis and Brønsted active sites, respectively.

### 3.2. Catalytic Activity

**Figure 4:**
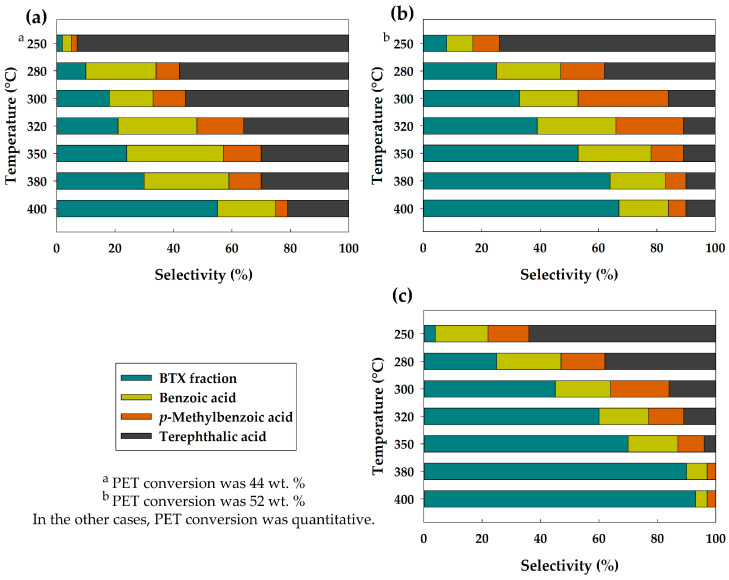
The effect of reaction temperature on the product selectivity under H_2_ pressure of (**a**) 5 MPa; (**b**) 7 MPa; (**c**) 9 MPa.

The catalytic activity of the Ni_2_P obtained in situ was investigated in PET hydroprocessing at 250–400 °C, under 5–9 MPa H_2_, during 6 h of reaction. PET conversion was quantitative in all cases, except for two. At 250 °C under 5 MPa H_2_, the conversion was 44 wt. %, and at 250 °C under 7 MPa H_2_, the conversion was 52 wt. %. An increase in pressure as well as an increase in temperature promotes the quantitative depolymerization of PET. As a result of PET depolymerization, monomers such as terephthalic acid and ethane are obtained. TPA production was confirmed via ^1^H, ^13^C NMR (Figures S2 and S3). Ethane formation was confirmed using gas chromatography (Table S3). TPA, in turn, undergoes subsequent transformations: direct deoxygenation (DDO) and decarboxylation (DCO_2_). Figure 4 shows the effect of temperature and pressure on the product selectivity. BTX fraction, and oxygen-containing compounds, such as TPA, *p*-methylbenzoic (*p*-MBA) and benzoic (BA) acids were obtained among the reaction products. It has been found that both an increase in temperature and in pressure contribute to an increase in the selectivity for the target BTX fraction and a decrease in the selectivity for terephthalic acid. TPA was not detected at all among the reaction products at 380 °C and 400 °C under 9 MPa. Following the dynamics of changes in the selectivity for *p*-MBA and BA, in most cases, with increasing temperature, the selectivity for them first increases and then decreases. It is related to the TPA conversion into these substances, and to their following transformation into BTX fraction. All transformations occurring with PET and its derivatives proceed over metal or acid active sites of the catalyst.

**Figure 5:**
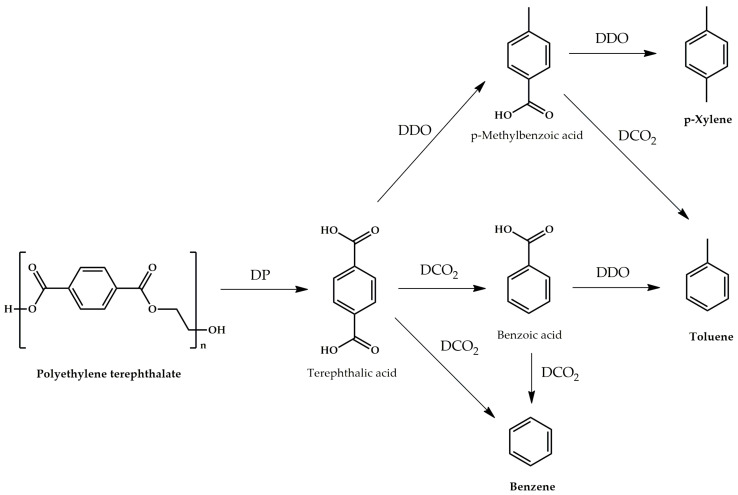
Proposed pathways of PET recycling into BTX fraction over Ni_2_P-containing catalyst.

Based on the experimental data obtained, reaction pathways were proposed (Figure 5). First, PET depolymerization with the formation of TPA and ethane occurred. Acidic catalysts are known to catalyze PET depolymerization with the TPA obtained [21]. Therefore, it can be concluded that acid sites of the Ni_2_P-based catalyst catalyze depolymerization reaction. Then, TPA can be transformed via three pathways. Through DDO, *p*-MBA acid is obtained. Benzoic acid and benzene are formed by DCO_2_ reaction. DDO occurs over acid sites, while DCO_2_ occurs over metal sites [22,23]. Aldehydes were not identified among the reaction products; thus, we suggest that BA and benzene are formed directly from TPA. From Figure 4, it can be concluded that DCO_2_ prevails over DDO, since in most cases, the selectivity for BA exceeds the selectivity for *p*-MBA. Benzene also can be formed as a result of BA DCO_2_; toluene can be formed as a result of *p*-MBA DCO_2_ and BA DDO; and *p*-xylene is formed only as a result of *p*-MBA DDO. Figure S4 shows that the total selectivity for toluene and benzene prevails over the selectivity for *p*-xylene, which indicates the predominance of DCO_2_ pathway over DDO. With an increase in temperature, the selectivity for DCO_2_ products increases, which correlates with an increase in the Ni^δ+^ species content on the catalyst surface.

In the present study, ring-hydrogenated products were not observed; this is an advantage of Ni_2_P-containing catalyst used compared to the ones described in the literature. Hongkailers et al. found that methylcyclohexane and dimethylcyclohexane are obtained in low amounts over Co/TiO_2_ catalyst [12]. Jing et al. made a comparison of noble metal-containing catalysts [9]. Ring-hydrogenated products, such as cyclohexane, methylcyclohexane, dimethylcyclohexane, cyclohexanecarboxylic acid, and 4-methylcyclohexanecarboxylic acid were obtained over Ru/Nb_2_O_5_, Pd/Nb_2_O_5_, Pt/Nb_2_O_5_, Ru/ZrO_2_, Ru/TiO_2_, Ru/HZSM-5. Moreover, only ring-hydrogenated products were obtained over Pt/Nb_2_O_5_. In other works, NiAl_2_O_4_ also was used as a catalyst for noble metals [10]. Cyclohexanecarboxylic acid, and 4-methylcyclohexanecarboxylic acid were obtained over Pd/NiAl_2_O_4_ and Ru/NiAl_2_O_4_. Thus, Ni_2_P-containing catalyst obtained in situ is an effective catalyst for PET recycling into BTX fraction.

### 3.3. Recycling Tests

**Figure 6:**
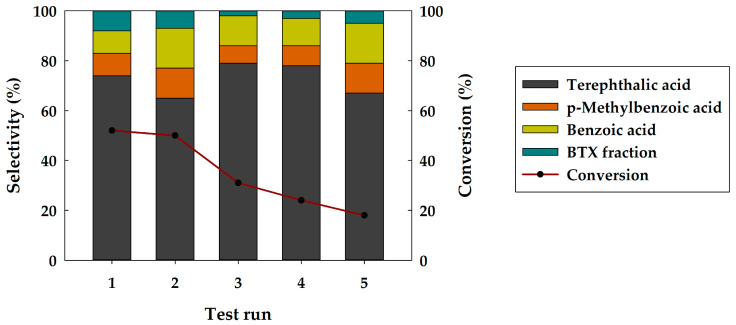
The recycling test of Ni_2_P-containing catalyst.

The possibility of catalyst reuse has been investigated within 5 runs (Figure 6). The recycling test runs of Ni_2_P were carried out under moderate PET conversion (250 °C, 7 MPa H_2_, 6 h). The choice of reaction conditions for the recycling test is determined by the fact that, at quantitative conversion or close to it, a possible deactivation of the catalyst may not be noticed. Hence, the conditions when the BTX selectivity was the highest at moderate conversion were chosen. After the 1st run, the conversion was 52 wt. % and almost did not change after the 2nd run. However, after the 3rd run of the reaction, the conversion gradually decreased and after the 5th run it was 18 wt. %. Product selectivity changed slightly during test runs.

**Figure 7:**
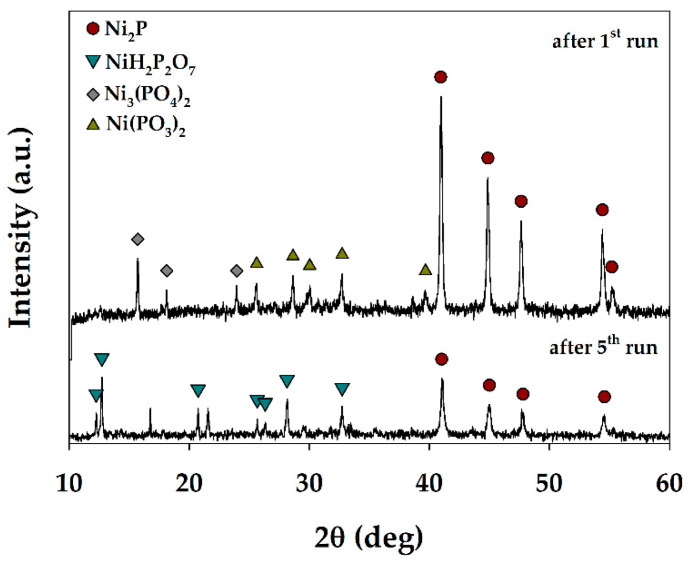
XRD patterns of the spent catalysts obtained at 250 °C, 7 MPa H_2_, 6 h after the 1st and 5th recycling test run.

The catalysts obtained after the 1st and the 5th run were investigated using the XRD technique (Figure 7). After the 1st run, Ni_2_P phase (PDF №74-1385) with the average crystallite size of 30 ± 2 nm was obtained. In addition, two phosphate phases, Ni_3_(PO_4_)_2_ (PDF №35-987) and Ni(PO_3_)_2_ (PDF №28-708), with the average crystallite sizes of 64 ± 14 nm and 32 ± 5 nm, respectively, were identified. After the 5th run, Ni_2_P and NiH_2_P_2_O_7_ (PDF №34-617) phases with average crystallite sizes of 29 ± 2 nm and 61 ± 11 nm, respectively, were observed. Thus, the average crystallite size of Ni_2_P remained unchanged. However, the composition of the phosphate phases has changed. Moreover, the peak intensity of each phase in the XRD pattern is known to depend on its volume content in the mixture. As a result, it can be assumed that the content of the Ni_2_P phase decreases after the 5th test run compared to the 1st one, which could be the reason for a decrease in the catalyst activity. However, other reasons of the catalyst deactivation, and the stability improvement remain the goals of further study.

## 4. Conclusions

Nickel phosphide catalysts were obtained in situ in the reaction medium during PET waste recycling. The presence of Ni_2_P phase in the catalysts was confirmed using XRD and HRTEM. It was shown via XRD that an increase in reaction temperature and pressure has almost no effect on the phase composition. However, an increase in temperature promotes crystallite growth, which can be a result of sintering. Nickel phosphate was also obtained using XRD and XPS. The Ni_2_P-containing catalyst obtained was applied in the PET to BTX thermocatalytic conversion for the first time. An increase in reaction temperature and pressure promotes an increase in BTX selectivity. The highest selectivity of 93% for BTX fraction was shown to be obtained at quantitative conversion. Then, the possibility of catalyst reuse was investigated. The PET conversion was found to decrease after the 3rd run, which might be related to the decrease in the Ni_2_P phase content in the catalyst sample. The investigation of other reasons for Ni_2_P-based catalyst deactivation, and the catalyst stability improvement are the goals of further studies.

## Data Availability

Not applicable.

## Supplementary Materials

The following supporting information can be downloaded at: https://www.mdpi.com/article/10.3390/polym15102248/s1, Table S1: The average crystallite sizes of the catalyst sample phases calculated using the Scherrer equation; Table S2: Oxidation states (Ni2*p*, and P2*p* regions) on the Ni_2_P surface; Table S3: The results of gaseous carbon-containing product analysis; Figure S1: High-angle annular dark field–scanning transmission electron microscopy (HAADF–STEM) images and EDX elemental mapping of the Ni_2_P-containig catalyst obtained in situ at (**a**) 300 °C; (**b**) 340 °C; (**c**) 380 °C; Figure S2: Part of the ^1^H NMR spectrum of terephthalic acid. 400 MHz, DMSO-d_6_; Figure S3: Part of the ^13^C NMR spectrum of terephthalic acid. 100 MHz, DMSO-d_6_; Figure S4: The effect of reaction temperature on the product selectivity under H_2_ pressure of (**a**) 5 MPa; (**b**) 7 MPa; (**c**) 9 MPa.
