# Supplementary material for: PET Waste Recycling into BTX Fraction Using In Situ Obtained Nickel Phosphide
Source: Polymers (Basel). 2023 May 10;15(10):2248. doi: 10.3390/polym15102248 (PMC10222822; doi:10.3390/polym15102248)
Supplement: Supplementary file 1 [file polymers-15-02248-s001.zip › polymers-2352692-supplementary.pdf]

PET Waste Recycling into BTX Fraction Using In Situ Obtained Nickel Phosphide

Maria Golubeva *, Mariyam Mukhtarova, Alexey Sadovnikov and Anton Maximov

A.V.Topchiev Institute of Petrochemical Synthesis, Russian Academy of Sciences (TIPS RAS), Moscow 119991, Russia

* Correspondence: vinnikova@ips.ac.ru

Table S1. The average crystallite sizes of the catalyst sample phases calculated using the Scherrer equation.

Reaction conditions	Phase	Average crystallite size (nm)
300 °C, 9 MPa H_2	$NiH_2P_2O_7$	41±3
	Ni_2P	25±1
340 °C, 9 MPa H_2	$NiH_2P_2O_7$	46±6
	Ni_2P	28±1
380 °C, 9 MPa H_2	$Ni(PO_3)_2$	64±6
	Ni_2P	41±6
340 °C, 5 MPa H_2	$NiH_2P_2O_7$	46±6
	Ni_2P	30±3
340 °C, 7 MPa H_2	$NiH_2P_2O_7$	40±5
	Ni_2P	30±1

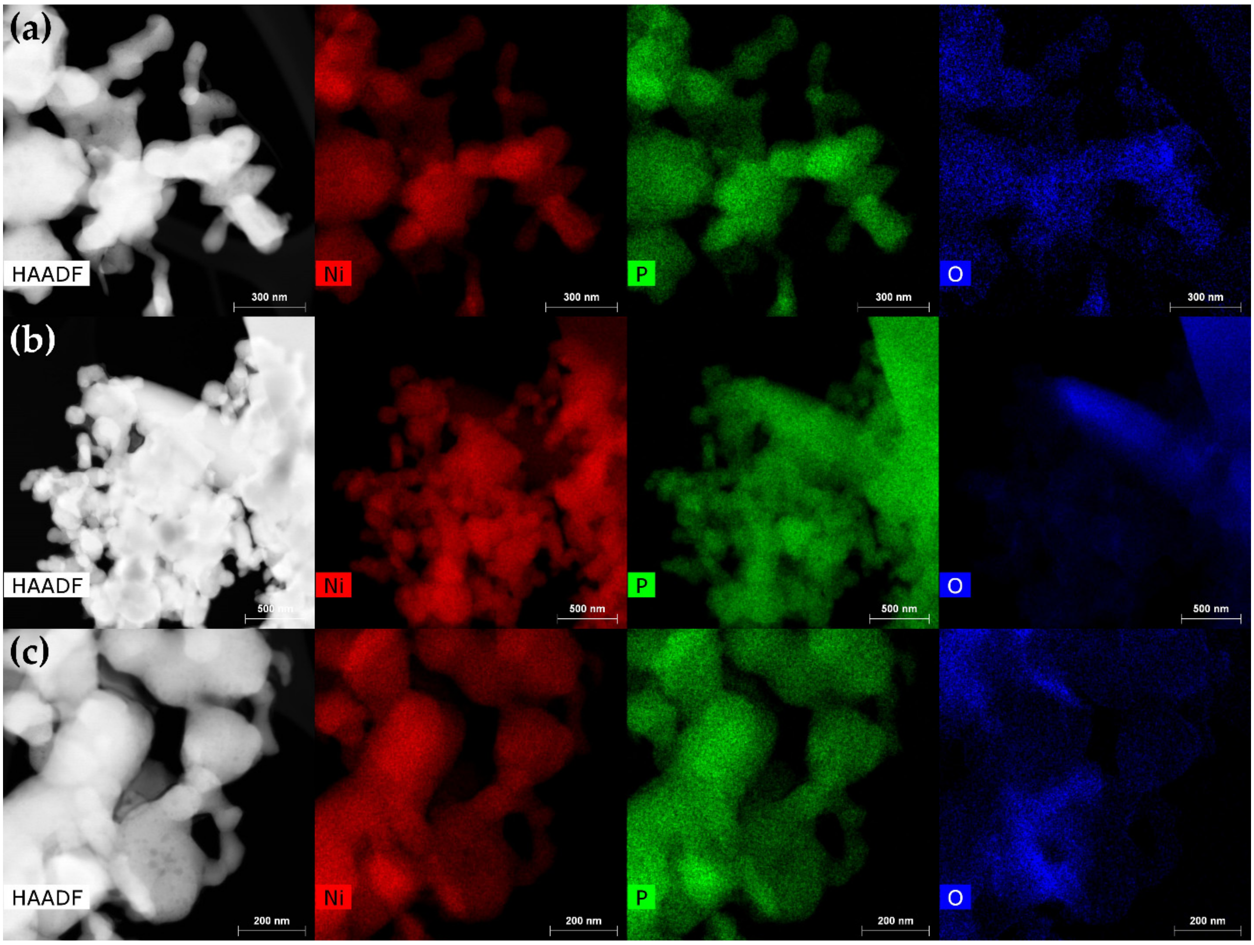

Figure S1. High-angle annular dark field–scanning transmission electron microscopy (HAADF–STEM) images and EDX elemental mapping of the Ni_2P-containig catalyst obtained *in situ* at (**a**) 300 °C; (**b**) 340 °C; (**c**) 380 °C.

Table S2. Oxidation states (Ni2*p*, and P2*p* regions) on the Ni_2P surface.

Temperature (°C)	State	Region	Binding energy (eV)	Content (%)
300	$Ni^{\delta+}$	$2p_{3/2}$	853.8	1.6
	Ni^{2+}		857.0	57.0
	shake-up satellite		862.3	41.4
	P^{5+}	$2p_{3/2}$	135.0	63.6
		$2p_{1/2}$	136.6	36.4
340	$Ni^{\delta+}$	$2p_{3/2}$	853.4	6.3
	Ni^{2+}		857.0	58.2
	shake-up satellite		862.0	35.5
	P^{5+}	$2p_{3/2}$	134.2	65.7
		$2p_{1/2}$	135.7	34.3
380	$Ni^{\delta+}$	$2p_{3/2}$	853.1	8.8
	Ni^{2+}		857.0	57.7
	shake-up satellite		861.7	33.5
	P^{5+}	$2p_{3/2}$	135.2	71.3
		$2p_{1/2}$	136.4	28.7

Characterization of terephthalic acid by NMR

^{1}H NMR (400 MHz, DMSO-d$_6$): δ = 8.04 (s, 4H; ArH), δ = 13.29 (s, 2H; COOH).

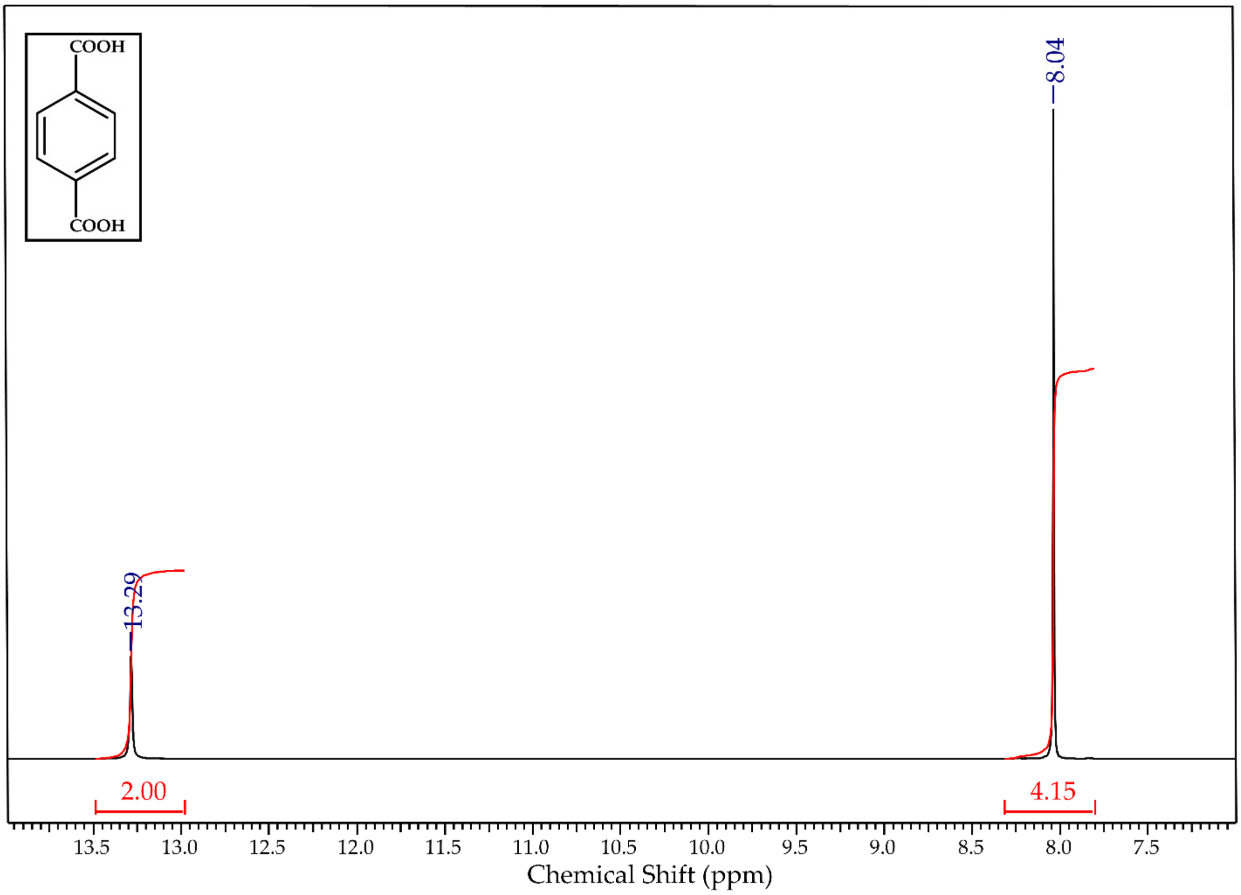

Figure S2. Part of the ^{1}H NMR spectrum of terephthalic acid. 400 MHz, DMSO-d$_6$.

^{13}C NMR (100 MHz, DMSO-d_6): δ = 166.67 (COOH), δ = 134.45, 129.47 (CAr).

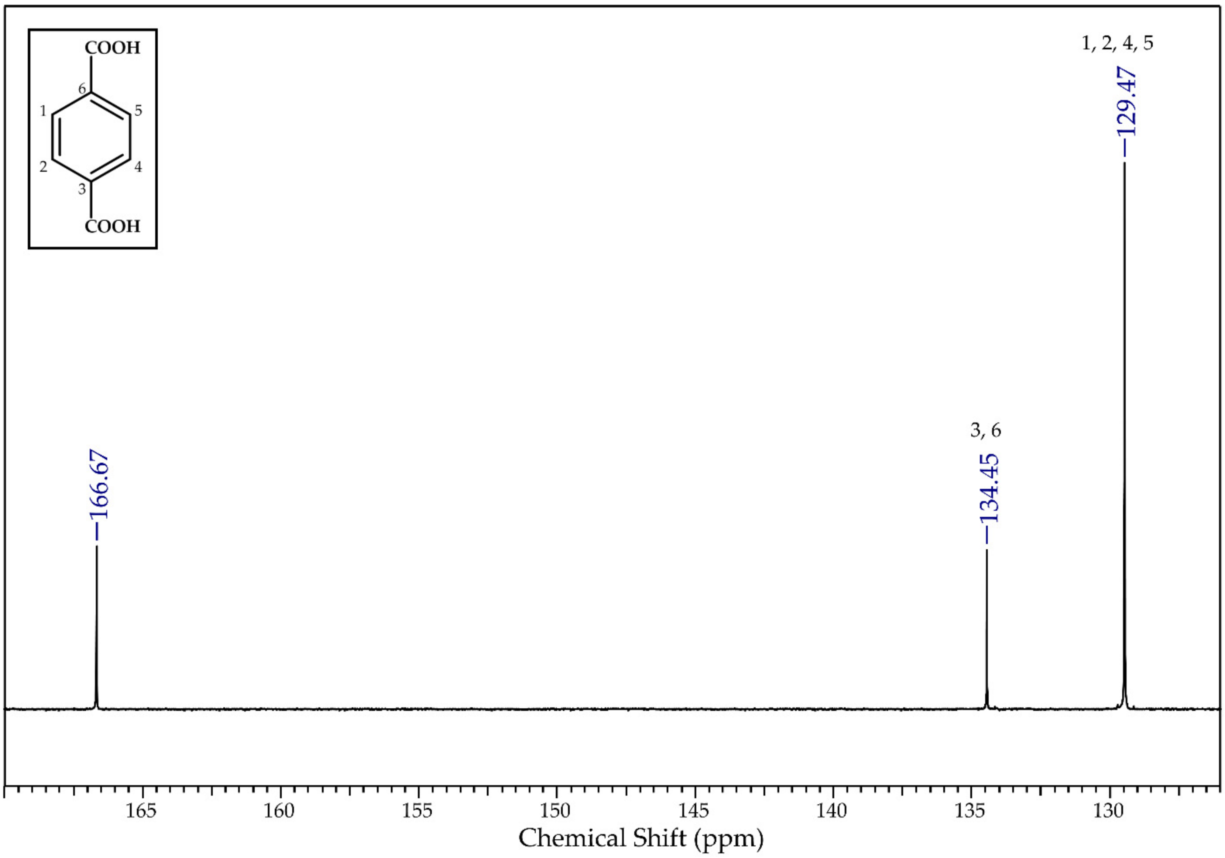

Figure S3. Part of the ^{13}C NMR spectrum of terephthalic acid. 100 MHz, DMSO-d_6.

Table S3. The results of gaseous carbon-containing product analysis.

**Gases**	**φ (%)**
C_2H_6	0.244
CH_4	traces
CO	0.110
CO_2	traces

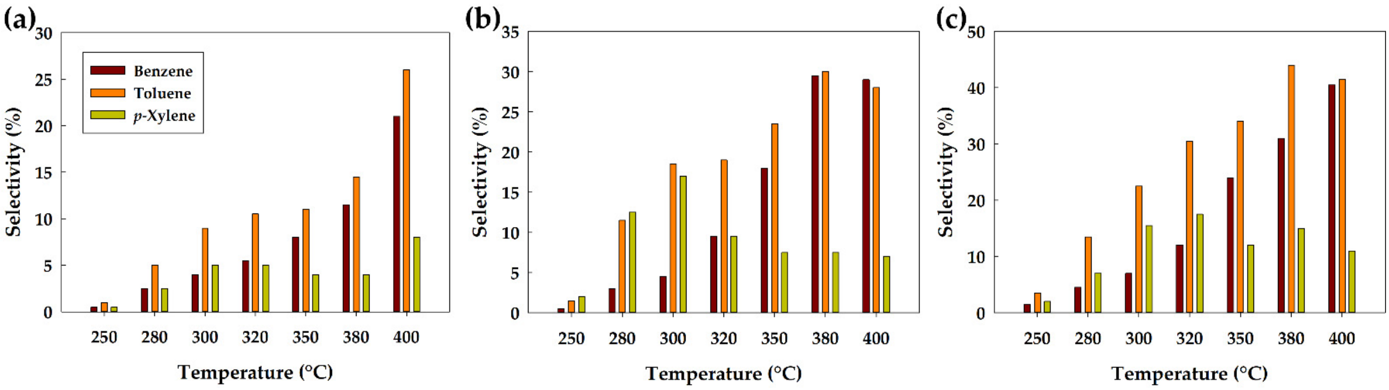

Figure S4. The effect of reaction temperature on the product selectivity under H_2 pressure of (**a**) 5 MPa; (**b**) 7 MPa; (**c**) 9 MPa.
